# Author Correction: MOADE: a multimodal autoencoder for dissociating bulk multi-omics data

**DOI:** 10.1186/s13059-025-03826-w

**Published:** 2025-10-09

**Authors:** Jiao Sun, Ayesha A. Malik, Tong Lin, Ayla Bratton, Yue Pan, Kyle Smith, Arzu Onar-Thomas, Giles W. Robinson, Wei Zhang, Paul A. Northcott, Qian Li

**Affiliations:** 1 Department of Biostatistics, St. Jude Children’s Research Hospital, Memphis, TN 38105 USA; 2 Department of Computer Science, University of Central Florida, Orlando, FL 32816 USA; 3 Department of Developmental Neurobiology, St. Jude Children’s Research Hospital, Memphis, TN 38105 USA; 4 Department of Oncology, St. Jude Children’s Research Hospital, Memphis, TN 38105 USA; 5 Center of Excellence for Neuro-Oncology Sciences, St. Jude Children’s Research Hospital, Memphis, TN 38105 USA

**Author correction: Genome Biol 26, 325 (2025)**

**https://doi.org/10.1186/s13059-025-03805-1**

Following publication of the original article [1], authors identified that Fig. 4 and Fig. 5 were erroneously transposed.

The correct order is given below and the original article [1] has been corrected.

**Fig. 4:**
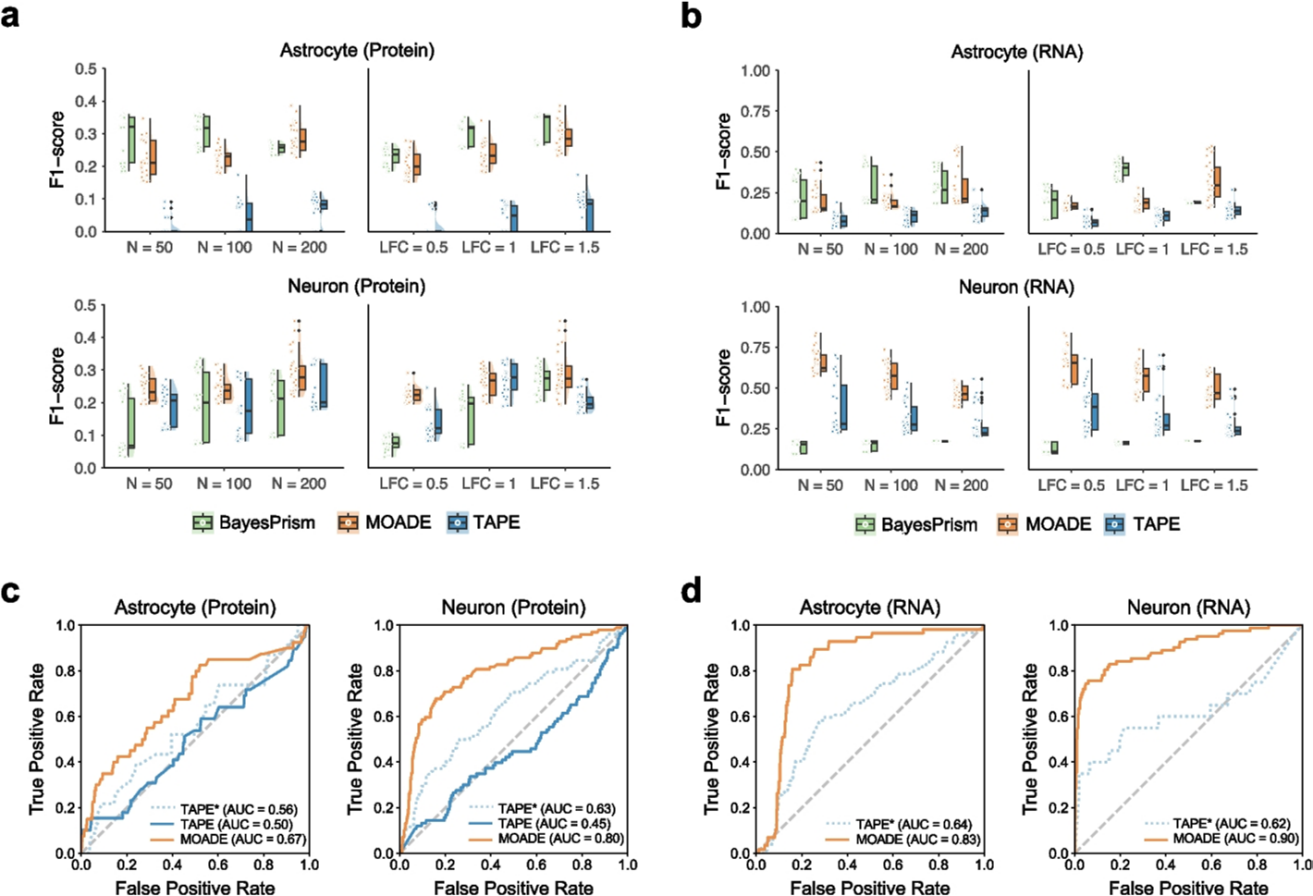
Cell-type-specific multiome profiles recover differentially expressed features. **a**, **b** Raincloud plots for F1-score on ctsDE detection in Astrocyte- and Neuron-specific proteome and transcriptome by MOADE, TAPE, and BayesPrism. **c**, **d** Area under the receiver operating characteristic curve (AUC) for the best F1-score of each method based on the same target data (*N* = 200, LFC = 1.5). Solid lines represent the autoencoder hyperparameter setting used in the best result of MOADE, which may or may not be identical to the default setting used in TAPE (dotted line)

**Fig. 5:**
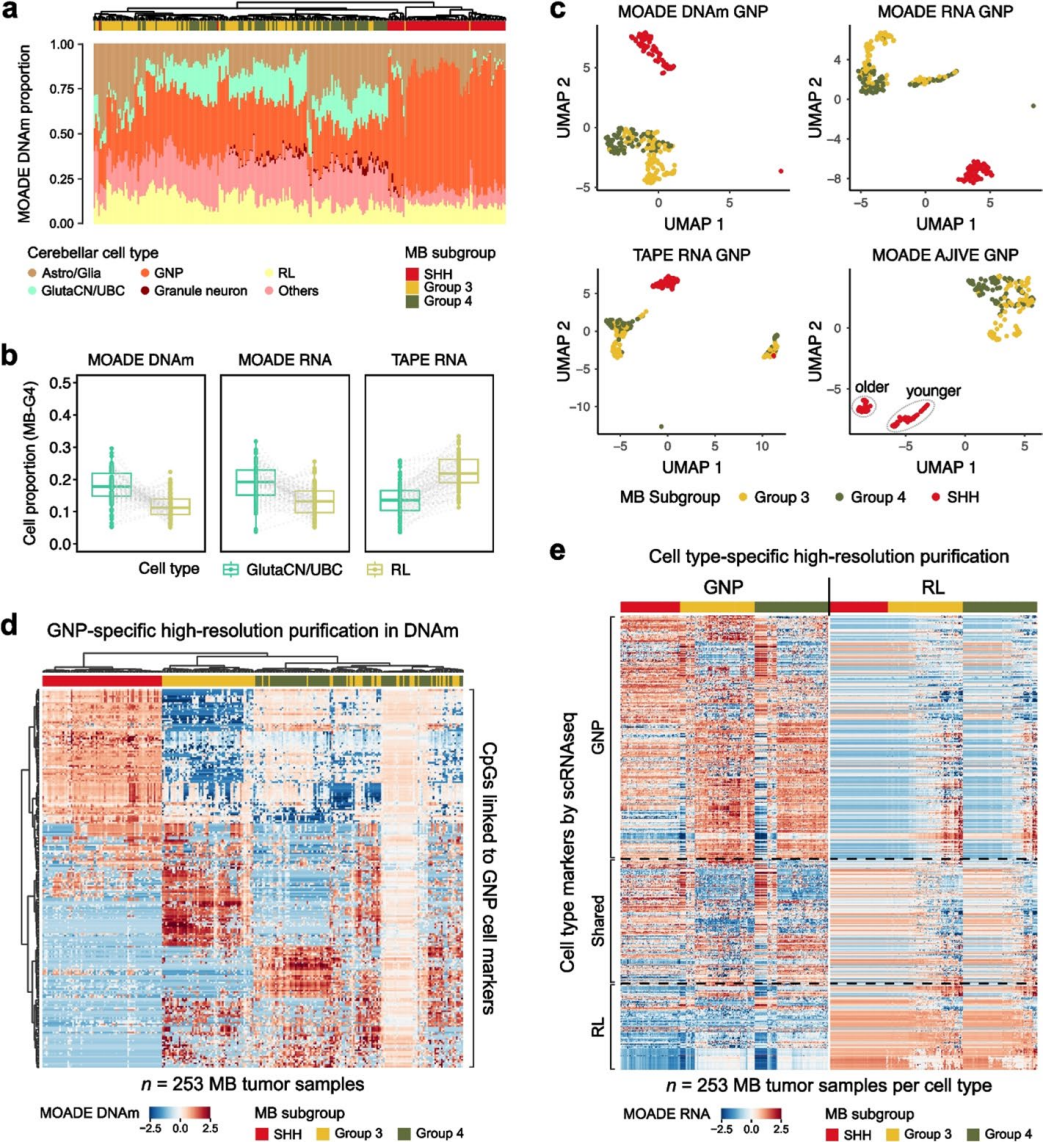
Multi-omic dissociation of pediatric medulloblastoma tumors with human fetal cerebellum scRNA-seq reference. **a** Origin cell proportions in bulk MB methylomes quantified by MOADE. **b** MOADE (DNAm, RNA) and TAPE (RNA) proportions of GlutaCN/UBC and RL in G4 MB tumors. **c** UMAPs of GNP-specific high-resolution profiles purified by MOADE in DNAm, RNA and by TAPE in RNA; UMAP of AJIVE-integrated GNP-specific multiomes purified by MOADE. **d**–**e** MOADE-predicted MB tumor DNAm (**d**) and gene expression (**e**) mapped to GNP or RL lineages for the marker genes identified in scRNA-seq data of human embryonal cerebellum
