# Supplementary material for: MOADE: a multimodal autoencoder for dissociating bulk multi-omics data
Source: Genome Biol. 2025 Sep 30;26:325. doi: 10.1186/s13059-025-03805-1 (PMC12487338; doi:10.1186/s13059-025-03805-1)
Supplement: Supplementary file 2 — Additional file 2 — Additional Figures and Tables. [file 13059_2025_3805_MOESM2_ESM.pdf]

MOADE: high-resolution digital dissociation with deep multimodal autoencoder

Additional File 2

Jiao Sun#, Ayesha A. Malik#, Tong Lin, Ayla Bratton, Yue Pan, Kyle Smith, Arzu Onar-Thomas, Giles W. Robinson, Wei Zhang, Paul A. Northcott, and Qian Li*

Contents

1 Developmental human brain RNA-seq and ATAC-seq **2**
1.1 Concordance of JNMF purified ATAC with scATAC-seq ground truth 2
1.2 Results of scSemiProfiler 2

2 In-silico gene expression and chromatin accessibility in glioblastoma tumors **4**

3 In-silico bulk transcriptomics and proteomics **6**

4 Application to pediatric medulloblastoma **8**

5 TCGA glioblastoma **13**

6 TCGA breast cancer **13**

1 Developmental human brain RNA-seq and ATAC-seq

1.1 Concordance of JNMF purified ATAC with scATAC-seq ground truth

To illustrate that JNMF (MOADE Step 1) does not fully recover cellular information from target tissues and does not lead to overfitting in MOADE Steps 2-3, we compared the output from JNMF with the ground truth cell proportions and cell-type-specific (CTS) accessibility profiles. The Pearson correlation coefficient (PCC) for JNMF prediction vs. ground truth cell proportions was < 0.6 for both ATAC-seq and RNA-seq, being worse than the final predition by MOADE autoencoder. Further, the ground truth CTS profile for each target pseudo bulk sample is the sum of peak counts per cell type. The input admixture ATAC data for JNMF was at log scale. Hence, the purified data was transformed back to the original scale (by exponential) to perform concordance evaluation. A large proportion of the purified values were highly correlated with ground truth, although there were inflated errors (or biases) in each sample. PCC for each sample are shown in each scatter plot below, while the PCC per cell type per sample is reported in Additional File 1 under spreadsheet 'JNMF_purified_ATAC_PCC'. The inflated scale of outliers in JNMF purified data does not affect the pre-training of MOADE autoencoder, because the pseudo bulk training samples were simulated based on the mean parameter of Poisson and then rescaled by min-max to the range of (0,1) before running autoencoder.

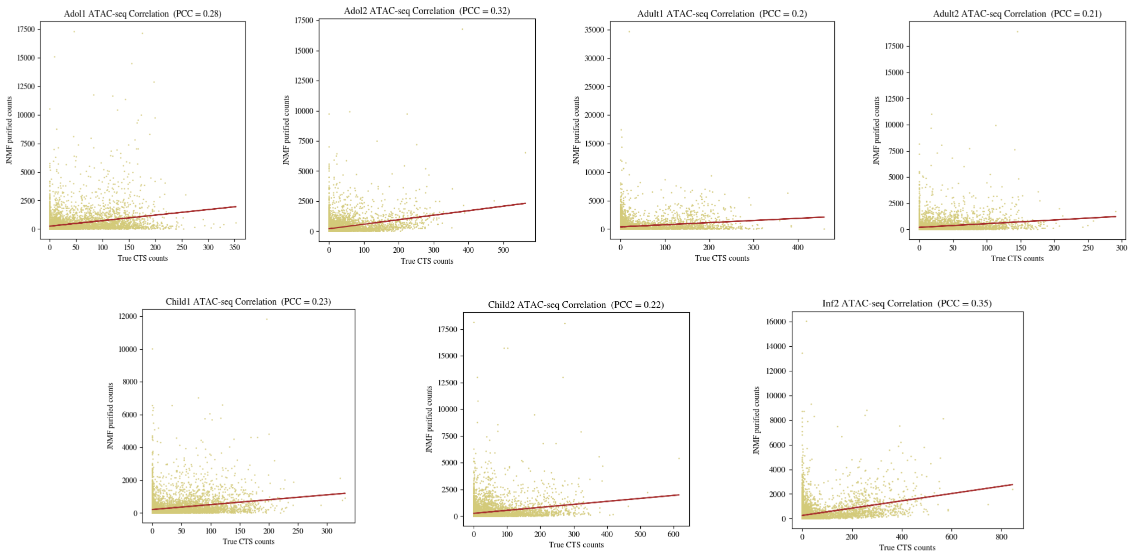

Fig. S1: **Pearson correlation for JNMF purified ATAC and ground truth CTS ATAC.**

1.2 Results of scSemiProfiler

The single cell reference samples and pseudo bulk target samples for running scSemiProfiler are described below. For the single cell data of n=2 fetal and n=1 infant neocortex samples, we randomly divided the cells per cell state into 20 groups and then constructed n=20 pseudo bulk samples by aggregating the cells per group. Next, the original n=7 pseudo bulk samples built from postnatal neocortex tissues were merged with the (in-silico) samples from Step 1 as a target cohort for deconvolution, whereas 20 out of 27 samples were overlapped between single cell reference and target bulk data. The predicted cell proportions of the $n = 7$ pseudo bulk samples not overlapping in single cell reference samples are visualized in scatter plots, with evaluation metrics shown in each plot.

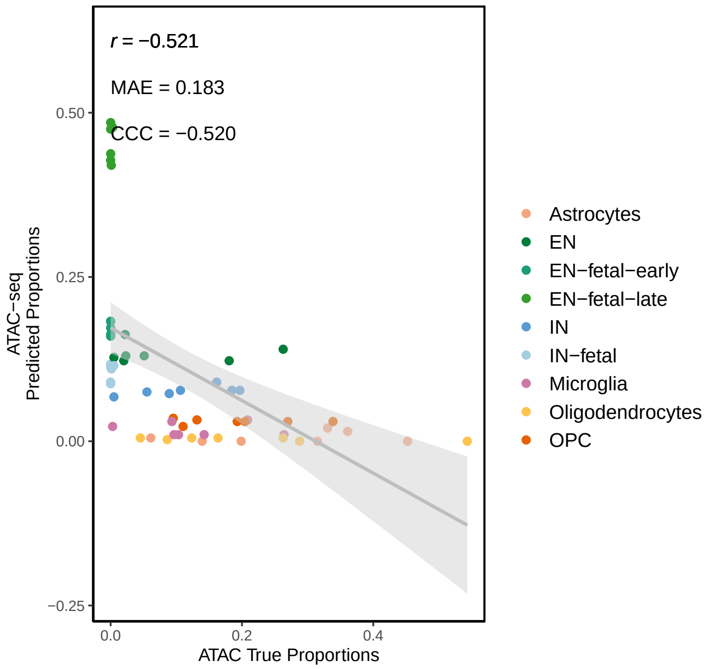

Fig. S2: **Scatter plot for scSemiProfiler deconvolution in pseudo bulk ATAC-seq.**

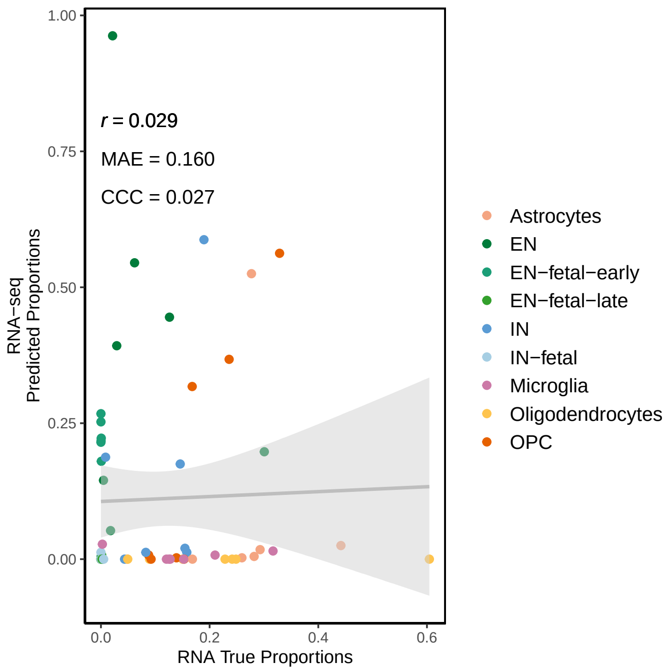

Fig. S3: **Scatter plot for scSemiProfiler deconvolution in pseudo bulk RNA-seq.**

2 In-silico gene expression and chromatin accessibility in glioblastoma tumors

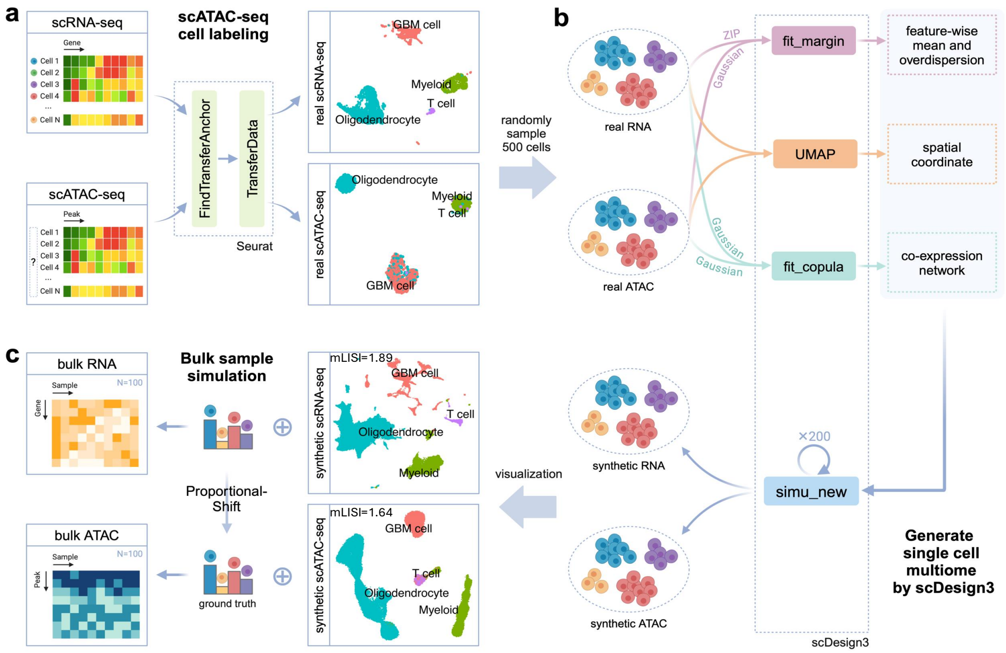

Fig. S4: **Simulation of RNA-seq and ATAC-seq paired by tumor tissues.** **(a)** Transfer scRNA-seq cell labels to scATAC-seq by Seurat. **(b)** Synthetic single cell multiome generation by scDesign3. **(c)** Simulated pseudo bulk gene expression and chromatin accessibility, with mean local inverse Simpson's Index (mLISI) shown in the UMAP.

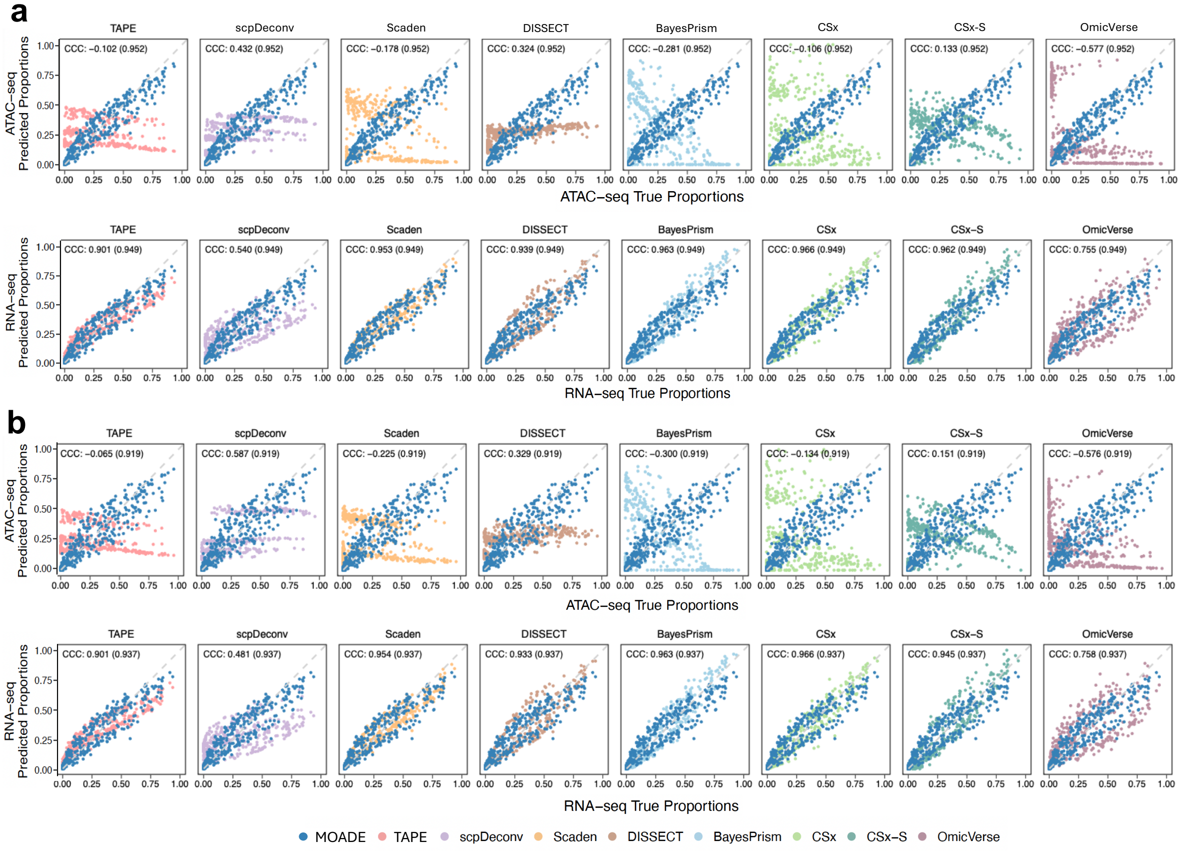

Fig. S5: **Performance of competing methods on in-silico RNA-seq and ATAC-seq paired by GBM tumors. (a, b)** Scatter plots of true (x axis) and predicted cell-type proportions (y axis) by deconvolving tumor simulated bulk gene expression and chromatin accessibility with either scRNA-seq or scATAC-seq from GBM tumor as reference. There are two scenarios representing small (**a**) and large (**b**) difference between molecular sources. The CCC between predicted proportion by each competing method and the ground truth is shown at the top of each plot, with MOADE result in parentheses.

3 In-silico bulk transcriptomics and proteomics

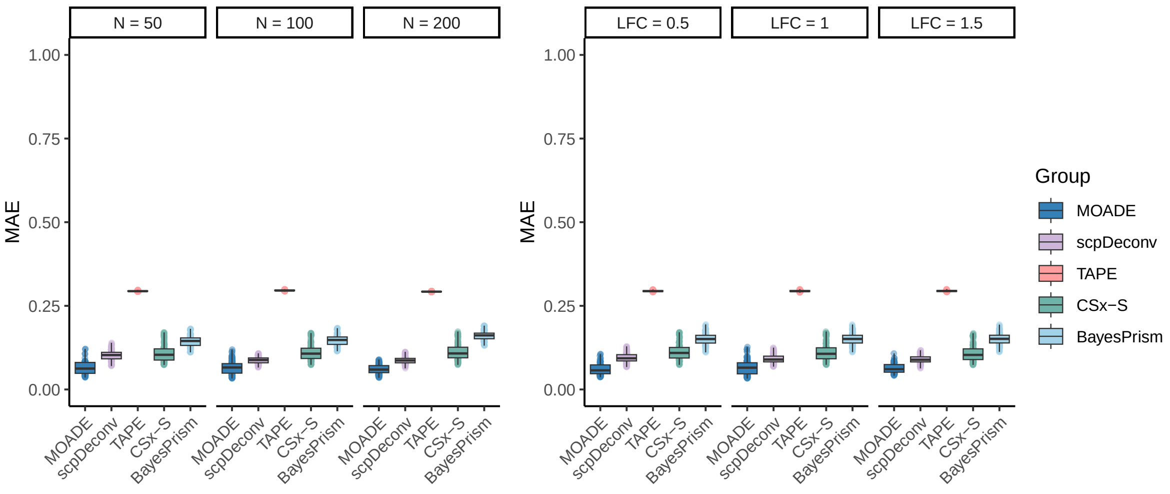

Fig. S6: MAE of MOADE, scpDeconv, TAPE, CSx-S, and BayesPrism on bulk proteomics deconvolution.

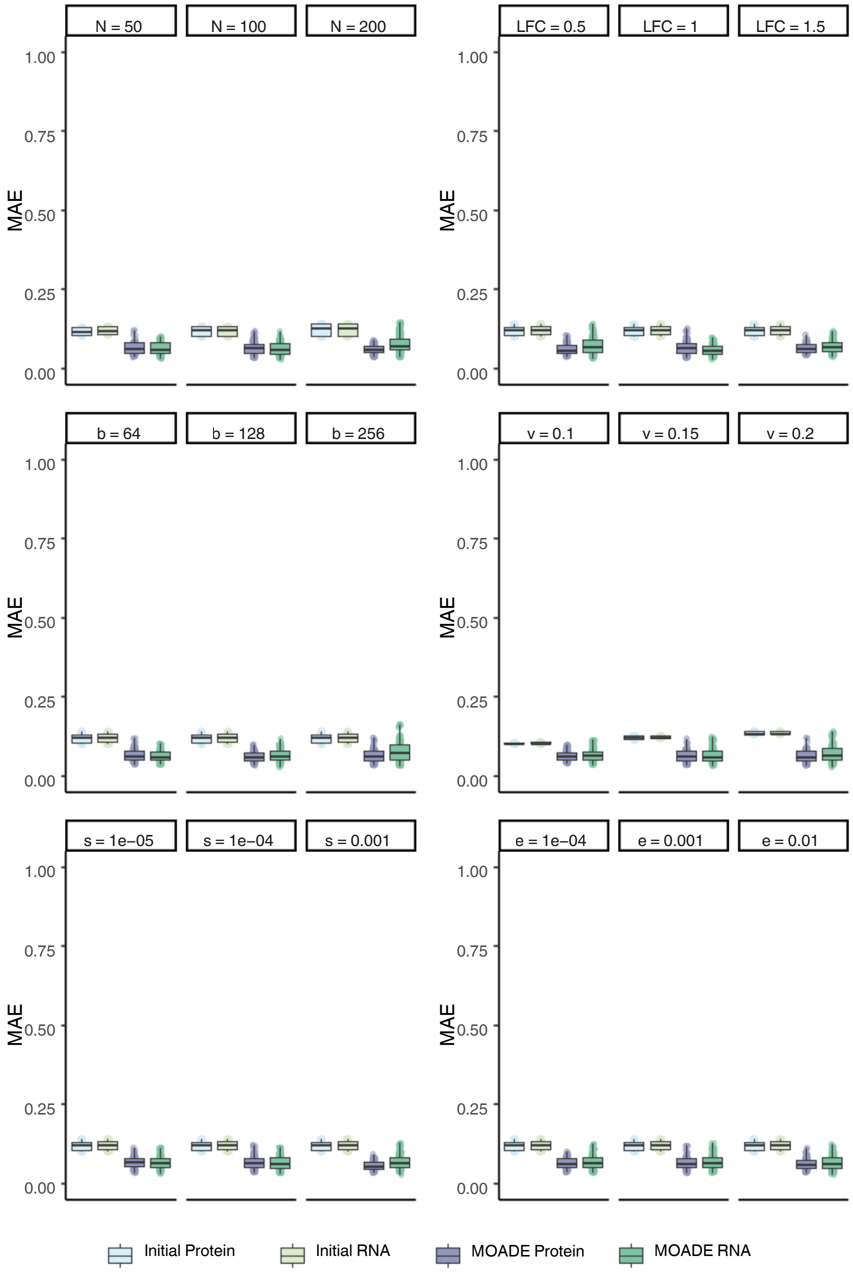

Fig. S7: MAE of MOADE and the initial cell count fractions in Step 1 JNMF purification by different normal tissue simulation scenarios: sample size (N), log fold change (LFC); and MOADE hyperparameters: batch size (b), initial value variance (v), step size (s), and convergence threshold (e).

4 Application to pediatric medulloblastoma

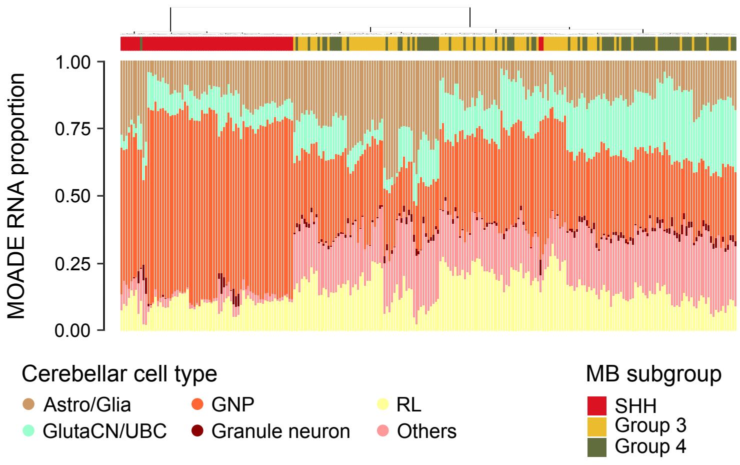

Fig. S8: Origin cell proportions in bulk MB transcriptomes resolved by MOADE.

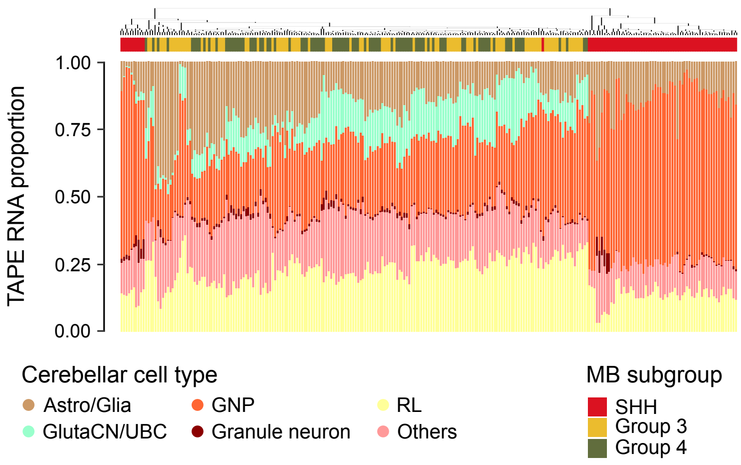

Fig. S9: Origin cell proportions in bulk MB transcriptomes resolved by TAPE.

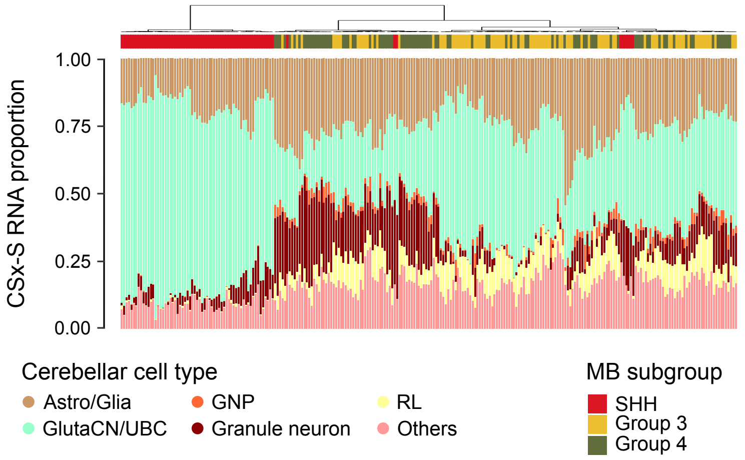

Fig. S10: Origin cell proportions in bulk MB transcriptomes resolved by CSx-S.

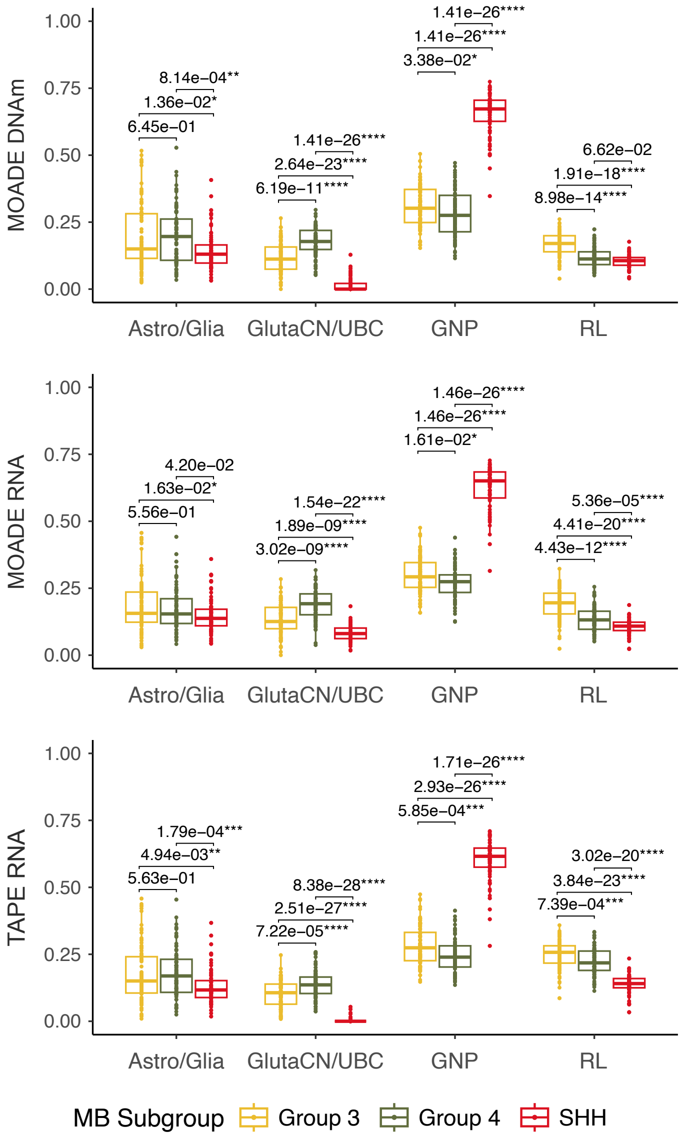

Fig. S11: Comparison of MOADE (DNAm, RNA) and TAPE (RNA) cell type proportions of Astro/Glia, GlutaCN/UBC, GNP, and RL in MB subgroups.

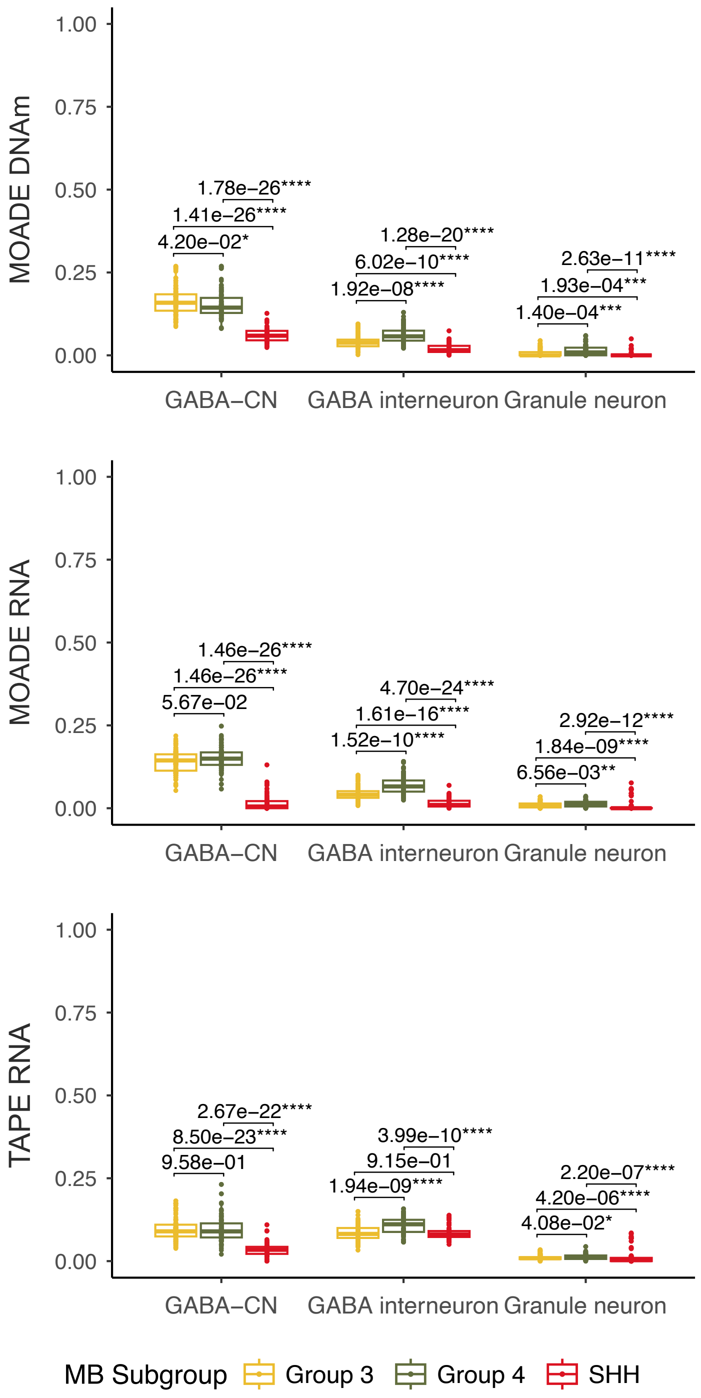

Fig. S12: Comparison of MOADE (DNAm, RNA) and TAPE (RNA) cell type proportions of GABA-CN, GABA interneuron, and Granule neuron in MB subgroups.

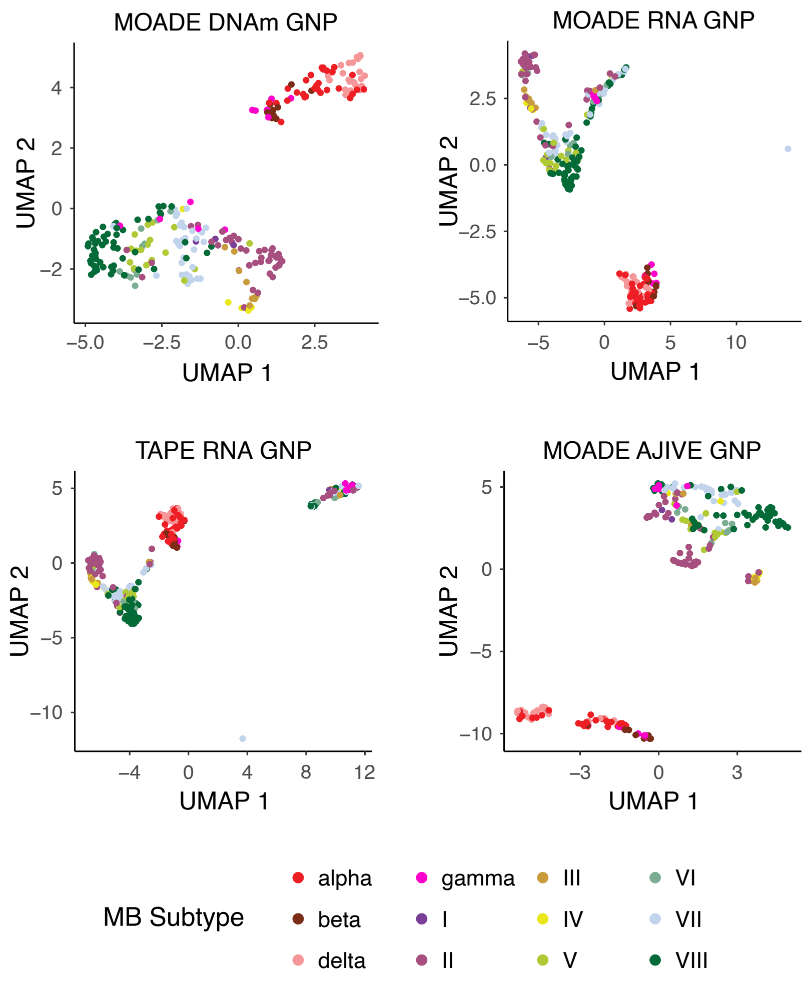

Fig. S13: UMAP of MOADE (DNAm, RNA), MOADE multiomes by AJIVE, and TAPE (RNA) purified profiles mapped to GNP cell state.

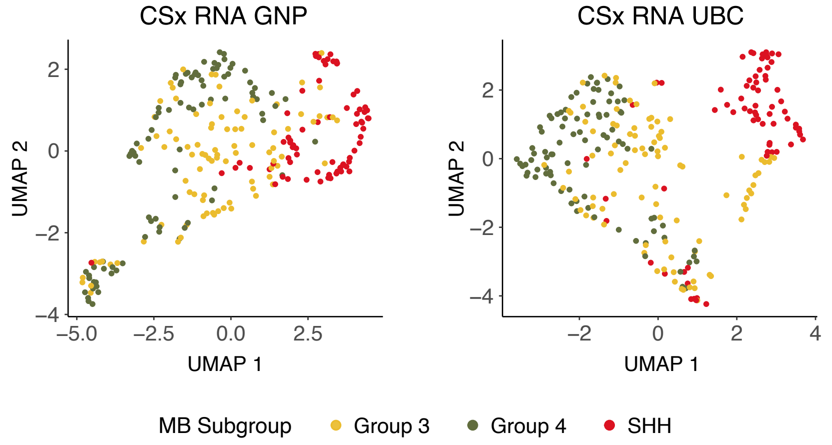

Fig. S14: UMAP of CSx RNA purified profiles mapped to GNP, UBC cell state.

5 TCGA glioblastoma

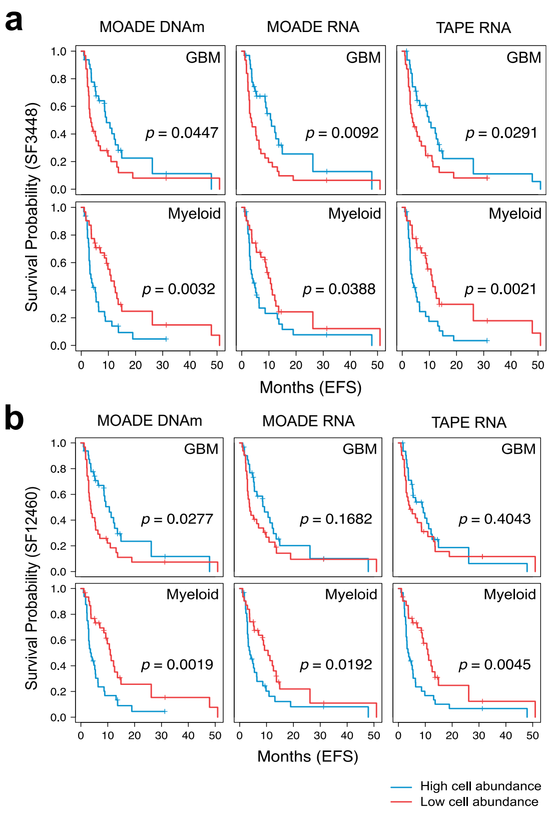

Fig. S15: **TCGA GBM patients' overall survival outcomes stratified by binarized cell proportions.** **(a)** Patient sample SF3448 serves as single cell reference. **(b)** Patient sample SF12460 serves as single cell reference.

		Event Free Survival				Overall Survival			
		GBM	Myeloid	Neuron	T cell	GBM	Myeloid	Neuron	T cell
SF3448	MOADE DNAm	**0.0447**	**0.0032**	0.6253	0.1359	0.5102	**0.0055**	0.2124	0.4130
	MOADE RNA	**0.0092**	**0.0388**	0.4257	0.6643	0.2438	0.1337	0.6881	0.8490
	TAPE RNA	**0.0291**	**0.0021**	0.9960	0.1367	0.2572	**0.0205**	0.8548	0.3819
SF12460	MOADE DNAm	**0.0277**	**0.0019**	**0.0222**	**0.0251**	0.3923	**0.0198**	**0.0115**	0.0944
	MOADE RNA	0.1682	**0.0192**	0.0968	0.9565	0.3004	0.1939	0.7678	0.8091
	TAPE RNA	0.4043	**0.0045**	0.0801	0.1676	0.8844	0.0801	0.1857	0.4293

Table S1: Log-rank p-value of TCGA GBM patients' event free and overall survival stratified by binarized cell proportions in GBM, Myeloid, Neuron, and T cell.

6 TCGA breast cancer

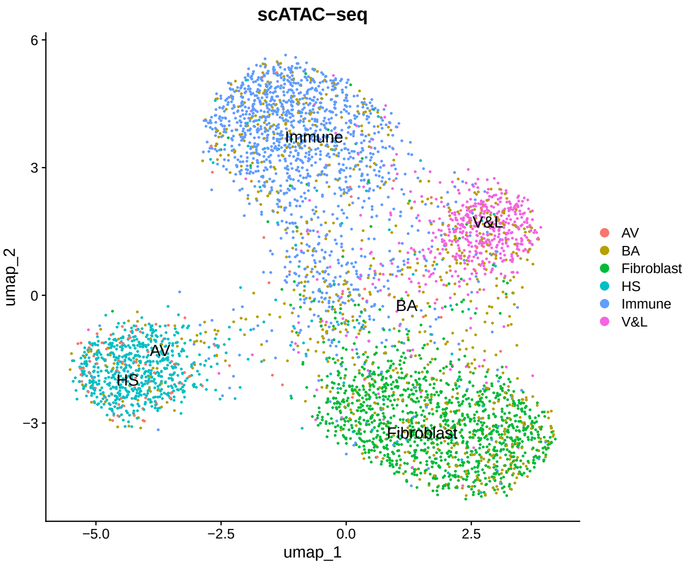

Fig. S16: **Human breast cancer scATAC-seq with original cell annotation determined by scRNA-seq.**

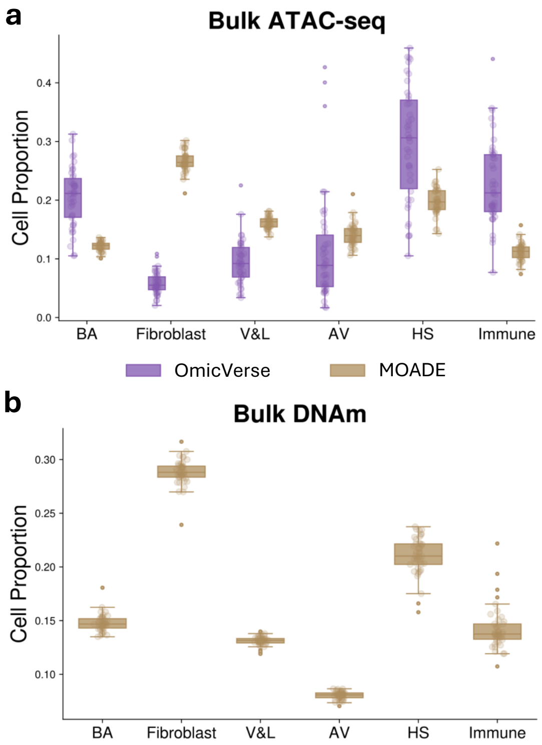

Fig. S17: **TCGA breast cancer TME composition using scATAC-seq reference with original cell annotation. (a)** TME composition in bulk ATAC-seq resolved by OmicVerse and MOADE. **(b)** TME composition in bulk DNAm resolved by MOADE.

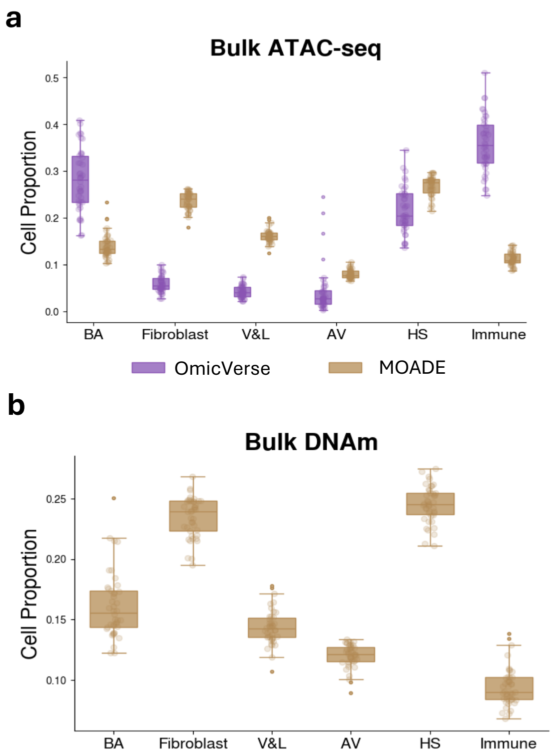

Fig. S18: **TCGA breast cancer TME composition using scATAC-seq reference with reannotation.** **(a)** TME composition in bulk ATAC-seq resolved by OmicVerse and MOADE. **(b)** TME composition in bulk DNAm resolved by MOADE.
